# nf-LO: A Scalable, Containerized Workflow for Genome-to-Genome Lift Over

**DOI:** 10.1093/gbe/evab183

**Published:** 2021-08-12

**Authors:** Andrea Talenti, James Prendergast

**Affiliations:** The Roslin Institute, University of Edinburgh, Midlothian, United Kingdom

**Keywords:** liftover, assembly, Nextflow, workflow

## Abstract

The increasing availability of new genome assemblies often comes with a paucity of associated genomic annotations, limiting the range of studies that can be performed. A common workaround is to lift over annotations from better annotated genomes. However, generating the files required to perform a lift over is computationally and labor intensive and only a limited number are currently publicly available.

Here we present nf-LO (nextflow-LiftOver), a containerized and scalable Nextflow pipeline that enables lift overs within and between any species for which assemblies are available. nf-LO will consequently facilitate data interpretation across a broad range of genomic studies.

##  

The advent of third-generation sequencing and ultrafast assemblers (Joseph et al. 2018; Ruan and Li 2020) allows for the generation of high-quality de novo assemblies in a fraction of the previous time. As a result, increasingly large numbers of new genomes for several species are being generated (Zoonomia Consortium 2020). Despite this increased availability, novel assemblies most often lack the extensive annotation data required to perform downstream analyses. Not only simple annotations such as gene models, but also supplementary resources for researcher to understand the biological significance of their studies. Unfortunately, such resources are generally only available for a small number of model organisms (Carithers et al. 2013; Amberger et al. 2015; Hu et al. 2019; OMIA 2020).

A solution to the problem is to lift over positions and annotations (i.e., cross-mapping of the loci) to the new genome from well-annotated assemblies, using tools such as LiftOver (Navarro Gonzalez et al. 2021) and NCBI Remap (Luu et al. 2020). However, the alignment files required to perform these analyses are not simple to generate and are therefore limited to a few popular reference genomes. For all other pairs of genomes researchers have to generate their own lift over files. Only a few algorithms address the problem in an easy to implement and distributable way, for example, flo for same species lift over (Pracana et al. 2017) and LiftOff for ultrafast lift over (Shumate and Salzberg 2021). In this study, we present nf-LO, a scalable workflow to generate lift over files for any pair of genomes based on the UCSC LiftOver pipeline. nf-LO can directly pull genomes from public repositories, supports parallelized alignment using a range of alignment tools and can be finely tuned to achieve the desired sensitivity, speed of process, and repeatability of analyses.

nf-LO is a workflow to facilitate the generation of genome alignment chain files compatible with the LiftOver utility. It is written in Nextflow, a domain-specific language and workflow manager that allows easy implementation, redistribution, and scalability of complex workflows across every Unix-based operating system; ranging from a desktop machine to cloud computing and HPC clusters. The dependencies are shipped alongside the workflow as docker containers or as an anaconda environment, facilitating the diffusion and adoption of the workflow across different systems.

The software accepts any two input genomes in fasta format, or alternatively can download a resource by providing a web address, an iGenome identifier or an NCBI GenBank or RefSeq accession. The workflow is shown in figure 1, and in brief consists of three core steps, and one optional one: 1) chunking the two genomes, 2) pairwise alignment of the blocks, 3) generating the chain-net file that can be used to perform the lift over and, if a bed/gff/gtf/vcf/bam/maf file is provided, 4) performing the lift over from source to target. The chunking approach dramatically reduces the runtime of the analysis by parallelizing the alignments.

**Fig. 1:**
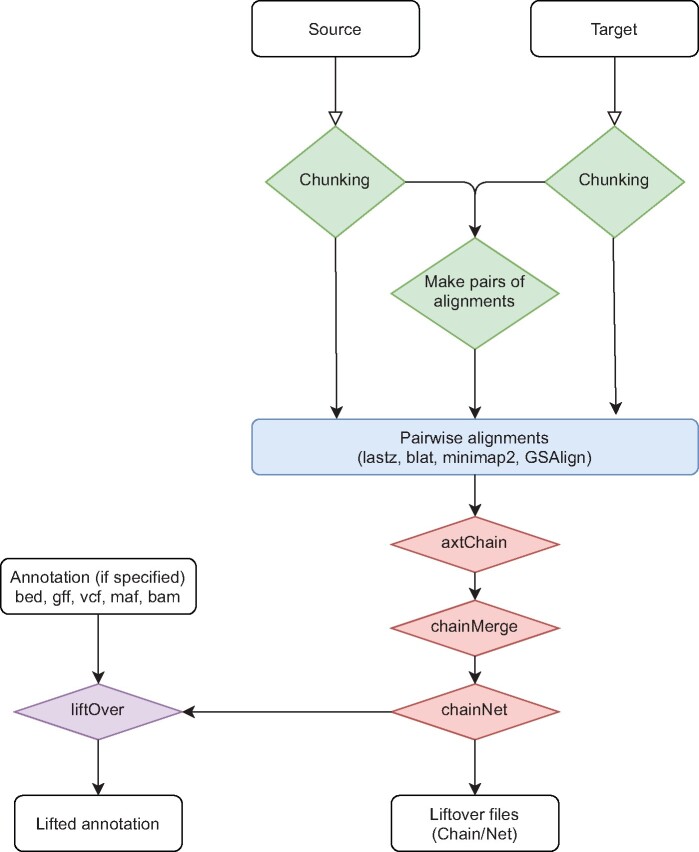
Scheme of the workflow of nf-LO with the chunking (step 1, in green), alignment (step 2, in blue), generation of the liftover files (step 3, in red), and optionally lifting of the variants to the target genome (step 4, in purple).

The alignment phase can be performed in different ways, depending on the type and sensitivity required by the user. For same-species alignments, we provide native support for both blat (Kent 2002), the aligner of choice for same species lift over files from the UCSC genome browser, and GSAlign (Lin and Hsu 2020), a new, high speed same-species alignment software. For performing different-species lift overs, nf-LO also incorporates lastz (Harris 2007), used by the UCSC genome browser to generate between species LiftOver files, and minimap2 (Li 2018), one of the fastest genome-to-genome aligners. All these aligners are integrated within the workflow, keeping unchanged the UCSC backbone for downstream stages (UCSC 2018). We provide canned configurations for each aligner based on how distant the two genomes are (e.g., near or far), with the possibility to provide sets of custom parameters to achieve the desired balance between speed and sensitivity (supplementary table 1, Supplementary Material online). nf-LO achieves similar lift over coverage as LiftOver files from UCSC with appropriate tuning of the parameters (supplementary table 2, Supplementary Material online).

The third stage processes the alignments analogously to the UCSC processing pipeline, obtaining the chain-net files to perform the actual lift over. Finally, the fourth step supports both the standard bed format with the LiftOver software, or several additional formats using CrossMap (Zhao et al. 2014), including popular formats such as VCF, BAM, and GFF. Optionally, the workflow can collect metrics on the lifted annotation when provided, as well as take advantage of mafTools (Earl et al. 2014) to report metrics for the chain file generated by the workflow. These metrics are then provided in HTML format to facilitate the interpretation and collection across multiple runs.

In conclusion, we provide a transposition of the UCSC lift over pipeline within the Nextflow language, together with the necessary containers to run the analyses, allowing an easy, streamlined implementation in any Unix-based system. We believe that this workflow will be of use across genomics studies, facilitating research work and enabling data interpretation.

## Supplementary Material

Supplementary data are available at *Genome Biology and Evolution* online.

## Supplementary Material

evab183_Supplementary_Data

## Data Availability

The code described in the article is publicly available on GitHub at the repository https://github.com/evotools/nf-LO, last accessed August 18, 2021. The documentation for the software can be accessed in the wiki page of the website (https://nf-lo.readthedocs.io, last accessed August 18, 2021).
